# Hepatoprotective effects of *Auricularia cornea* var. *Li*. polysaccharides against the alcoholic liver diseases through different metabolic pathways

**DOI:** 10.1038/s41598-018-25830-w

**Published:** 2018-05-15

**Authors:** Xiuxiu Wang, Yufei Lan, Yongfa Zhu, Shangshang Li, Min Liu, Xinling Song, Huajie Zhao, Weiru Liu, Jianjun Zhang, Shouxian Wang, Le Jia

**Affiliations:** 1 Institute of Plant and Environment Protection, Beijing Academy of Agriculture and Forestry Sciences, Beijing Engineering Research Center for Edible Mushroom, Key Laboratory of Urban Agriculture (North), Ministry of Agriculture, Beijing, P.R. China; 2 College of Life Science, Shandong Agricultural University, Taian, 271018 P.R. China; 3 Taian Academy of Agricultural Sciences, Taian, 271000 P.R. China; 4 The Second High School of Taian, Taian, 271018 P.R. China

## Abstract

The present work was designed to evaluate the antioxidation and hepatoprotective effects of *Auricularia cornea* var. *Li*. polysaccharides (APS) and enzymatic-extractable APS (EAPS) on the acute alcohol-induced alcoholic liver diseases (ALD). The *in vitro* antioxidant activities demonstrated that both APS and EAPS had strong reducing power and potential effects on scavenging reactive oxygen species. The *in vivo* mice experiments showed that the pretreatment with APS or EAPS showed potential hepatoprotective effects on the ALD possibly by increasing the antioxidant activities, reducing the lipid peroxidation, improving the alcohol metabolism, inhibiting the expression levels of inflammatory mediators and preventing the alcohol-induced histopathological alterations. In addition, the fourier-transform infrared (FT-IR), ^1^H and ^13^C nuclear magnetic resonance spectroscopy (NMR) and gas chromatography (GC) had been analyzed to obtained the primarily characteristics. The results indicated that abundant xylose and glucose contents probably had potential effects on possessing the bioactivities. The findings suggested that the *A. cornea* var. *Li*. might be considered as promising natural resource on exploring clinical drugs for the prevention and treatment with ALD and its complications.

## Introduction

As a complex organ with detoxifying functions on waste products, the liver plays important roles in metabolism of carbohydrate, protein and fat^1^. Alcohol consumption has a wide distribution in the world, and more than 40% of the population are consuming alcohol periodically^2^. However, the excessive alcohol consumptions daily can cause the alcohol liver damages, which is one of the most important factors inducing alcoholic liver diseases (ALD) and increasing the morbidity and mortality, increasingly aggravating the threaten on human health^3,4^. There are many lots of diseases and metabolic pathways are thought to be associated with ALD including steatosis, steatohepatitis, causing lipid accumulation, and up-regulation of cytochrome P4502E1 (CYP2E1), leading to oxidative stress and inflammatory injury^5,6^. It has been reported that the overproduction of reactive oxygen species (ROS) can induce oxidative stress, which is a deleterious process mediating the cellular damages, such as neurodegenerative disorder, cancer, cardiovascular diseases, atherosclerosis cataracts, and inflammation^7,8^. The increasing evidences have suggested that alcohol consumption can result in the enhancement of pro-inflammatory cytokines including interleukin-1β (IL-1β), tumor necrosis factor (TNF-α), and interleukin-6 (IL-6) *etc*., following by the up-regulation of inducible nitric oxide synthase (iNOS) and cyclooxygenase-2 (COX-2) levels^9,10^. Furthermore, oxidative stress and generation of free radicals play a critical role in the development of ALD^11,12^. From this point of view, effective antioxidant agents should be explored and synthesized against the ALD^13^. However, the synthetic antioxidants are toxic, and may induce serious side-effects in the long-time use clinically. Hence, it is worth believing that natural substances with superior antioxidant activities in inhibiting the oxidative-stress-induced damages have become attractive therapeutic strategies for the prevention and treatment of ALD and its complications^14^.

Natural polysaccharides extracted from edible-medicinal fungus, which are the most important and abundant substances not only in the fruiting-bodies but also in the mycelium, fermentation broth and residues, have been demonstrated to possess potential biological activities including antioxidant, antitumor, anti-inflammatory anticancer and immunological properties^15,16^. Thus, the polysaccharides can be regarded as new natural and safe compounds for applications in functional foods and medicine. *Auricularia cornea* var. *Li*., an evolutionary varietas of *A. auricula-judae*, has been received more and more attentions owing to its unique phenotype and potential medicinal properties^17^. However, scare papers about the antioxidant and hepatoprotective effects of polysaccharides extracted from the fruiting-bodies of *A. cornea* var. *Li*. against the ALD experimentally have been published up till now.

The objective of this work was to investigate the antioxidative and hepatoprotective effects of *A. cornea* var. *Li*. polysaccharides (APS) and enzymatic-APS (EAPS) on the alcohol-induced ALD, aiming to establish the possible hepatoprotective mechanism under oxidative stress.

## Results

### Monosaccharide composition analysis

**Figure 1:**
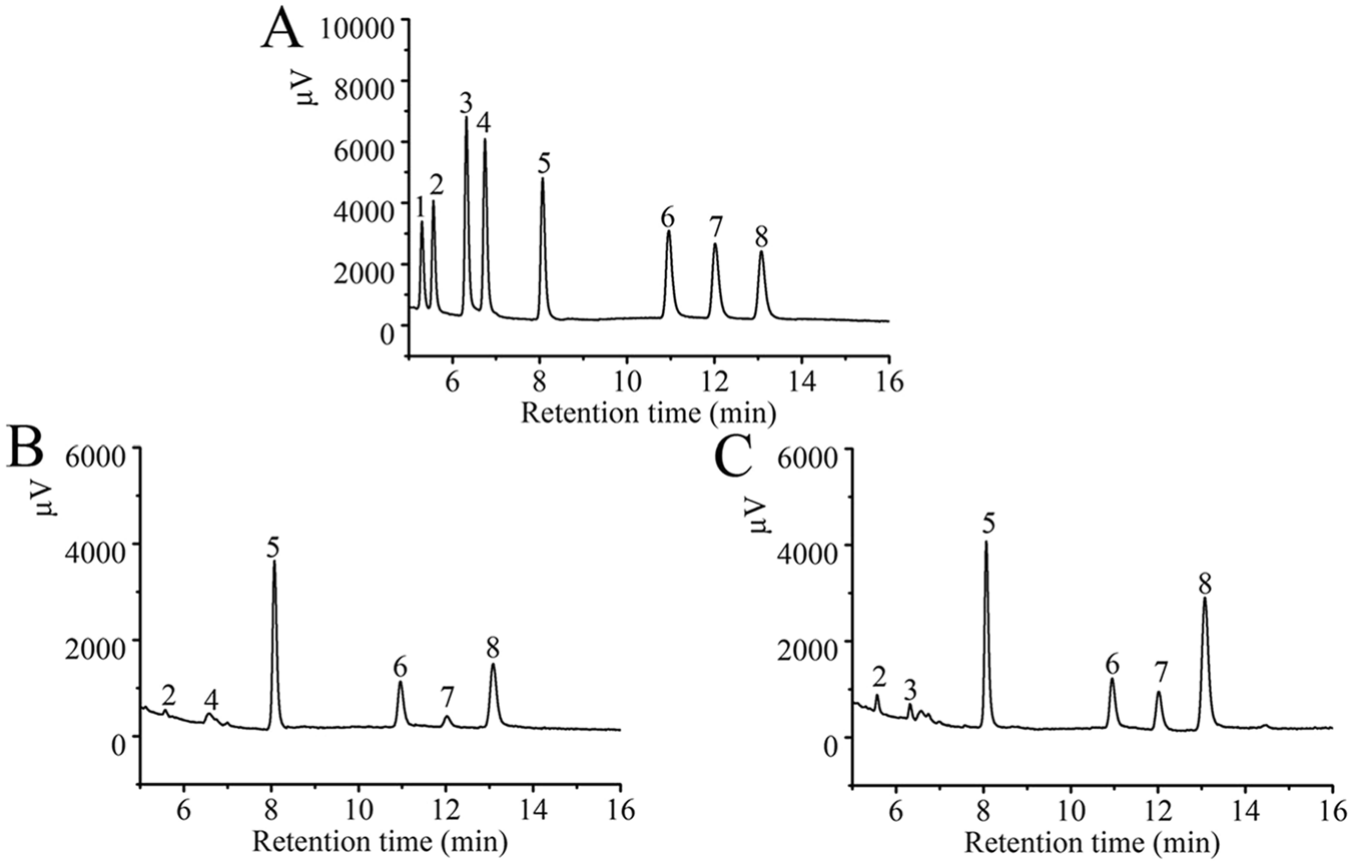
Gas chromatographs of (**A**) Standard monosaccharides, (**B**) APS and (**C**) EAPS. Peaks: (1) rhamnose, (2) fucose, (3) ribose, (4) arabinose, (5) xylose, (6) mannose, (7) galactose and (8) glucose.

The monosaccharide compositions of APS and EAPS were analyzed by means of gas chromatography (GC), and identified by comparison with the retention time of the standard sugars (Fig. 1A). The APS consisted six monosaccharides including fucose, arabinose, xylose, mannose, galactose and glucose with a percentage of 9.98%, 1.55%, 32.7%, 11.97%, 7.65% and 36.15% and a molar ratio of 5.9:1.0:21.1:6.4:2.3:53.7 (Fig. 1B), while EAPS composed of fucose, ribose, xylose, mannose, galactose and glucose with a percentage of 7.51%, 0.78%, 20.53%, 7.64%, 9.33% and 54.21% and a molar ratio of 8.8:1.0:26.4:8.2:10.0:58.1 (Fig. 1C), respectively.

### Characterizations of APS and EAPS

**Figure 2:**
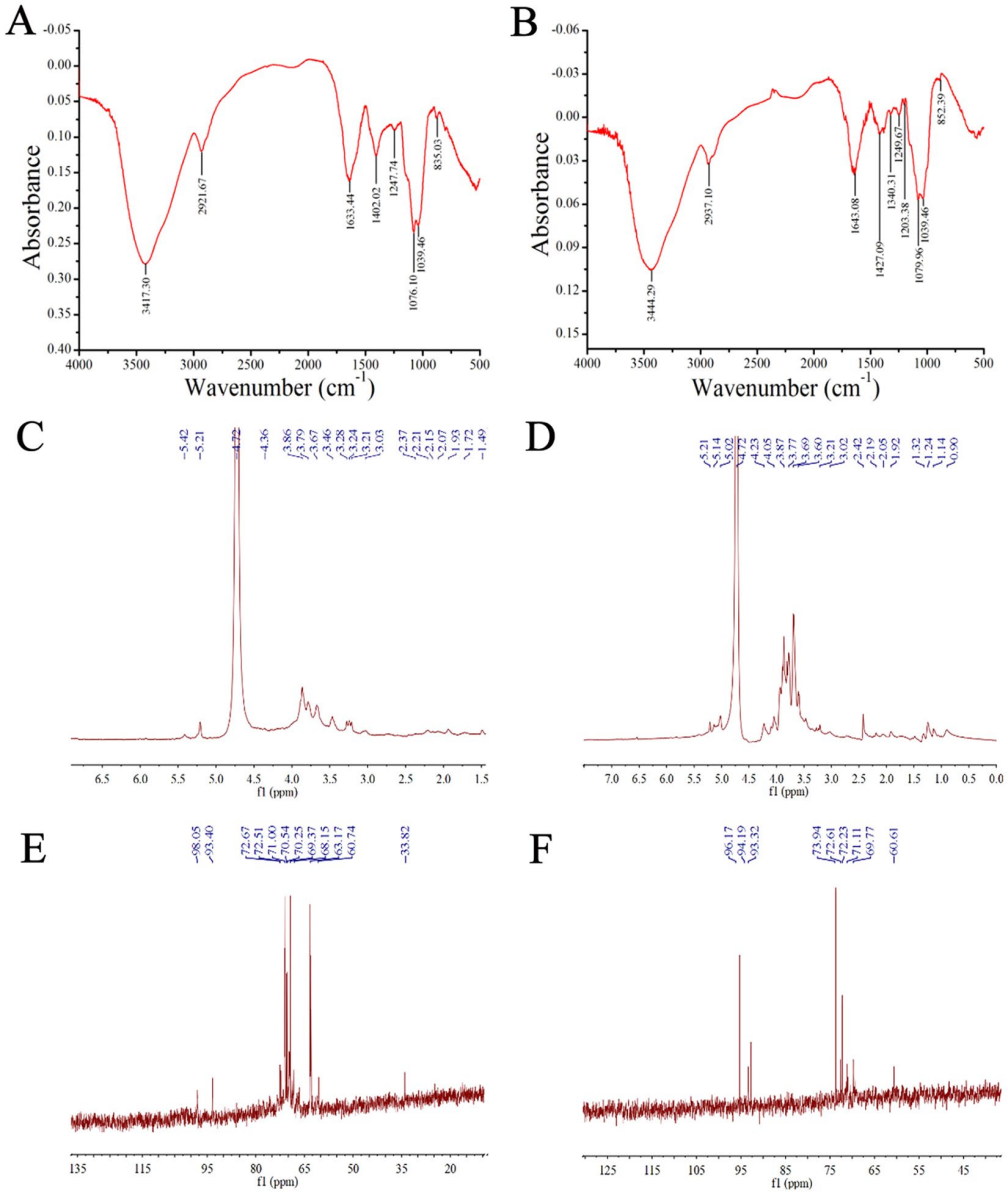
FT-IR spectra in the range of 4000–500 cm^−1^ of (**A**) APS, (**B**) EAPS, ^1^H NMR spectra of (**C**) APS, (**D**) EAPS and ^13^C NMR spectra of (**E**) APS, (**F**) EAPS.

The FT-IR spectra of APS and EAPS were depicted in Fig. 2A,B. As shown in the figures, the spectra of APS and EAPS showed typical and strong absorption peaks at 3417 and 3444 cm^−1^ for the –OH stretching vibrations, weak absorption peak at round 2921 and 2937 cm^−1^ for C–H stretching vibrations, 1633 and 1643 cm^−1^ for the stretching vibration of C=O, and the bands at about 1402 and 1427 cm^−1^ for –COOH variable angle vibrations, respectively, which indicated that both APS and EAPS revealed typical carbohydrate absorption peaks characteristics^18^. Furthermore, the strong characteristic absorption at 1040–1100 cm^−1^ was ascribed to sugar ring vibrations overlapping with stretching vibrations of C–O–C glycosidic bonds vibration, indicating the presence of pyranose in both polysaccharides^19^.

The ^1^H NMR spectra of APS and EAPS were shown in Fig. 2C,D. Two anomeric protons of APS at 5.21 and 5.42 ppm, as well as three anomeric protons of EAPS at 5.02, 5.14 and 5.21 ppm were obtained in this spectrum, demonstrating that APS and EAPS were corresponded to α-configuration^20^. In the anomeric region of ^13^C NMR spectrum (Fig. 2E,F), there were also two anomeric carbons of APS at 93.40 and 98.05 ppm, and three anomeric carbons of EAPS at 93.32, 94.19 and 96.17 ppm indicating the characteristic of the α-configuration, which were in accordance with the analysis of ^1^H NMR. Both ^1^H NMR and ^13^C NMR spectrum were well representative of carbohydrates^18^.

### *In vitro* antioxidant capacities

**Figure 3:**
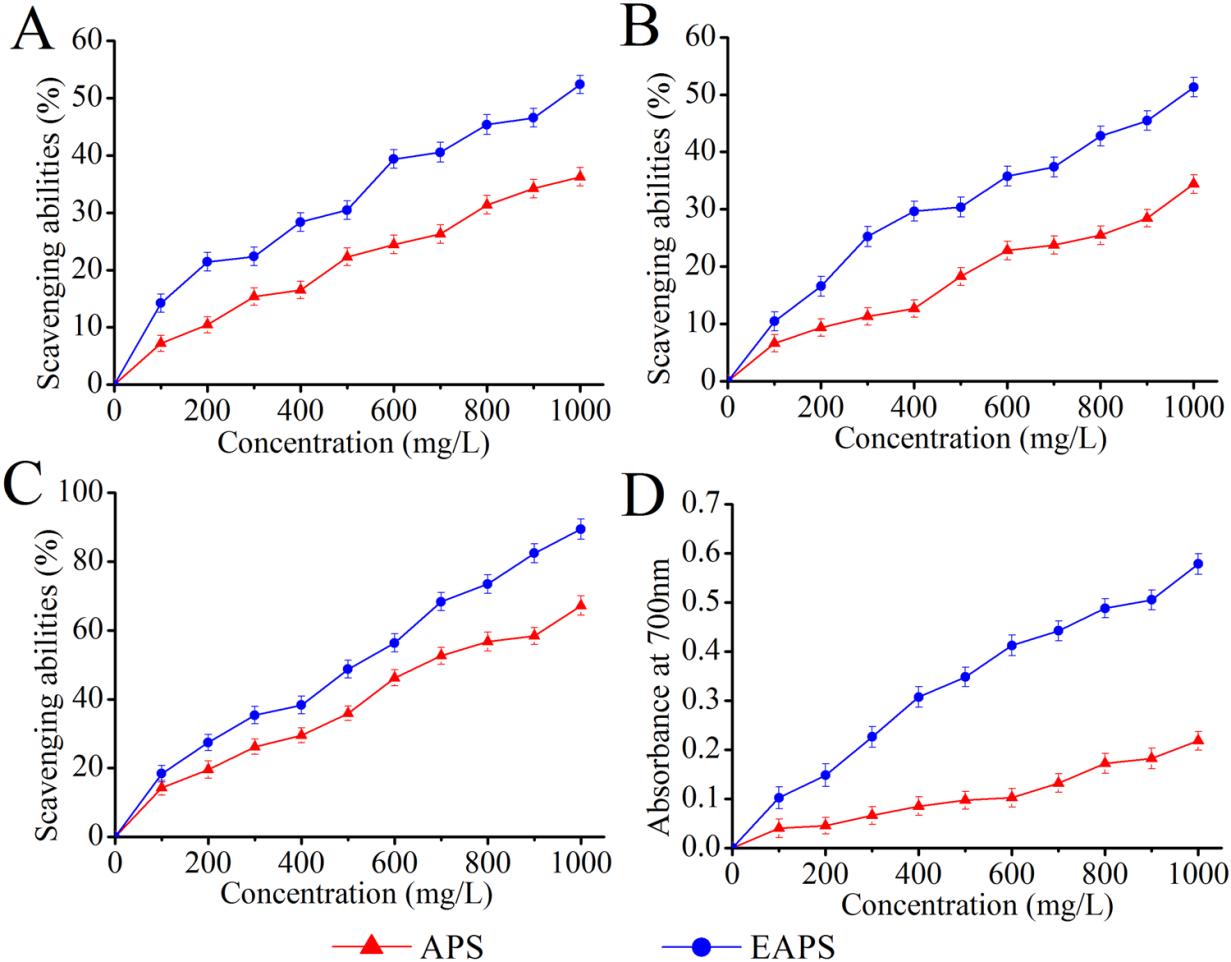
The *in vitro* antioxidant abilities of APS and EAPS. (**A**) Superoxide radicals, (**B**) hydroxyl radicals, (**C**) DPPH radicals and (**D**) reducing power.

The scavenging abilities on radicals were usually applied on the evaluation of antioxidant activities. As shown in Fig. 3, the scavenging abilities showed dose-dependent manners. At the concentration of 1000 mg/L, the scavenging abilities on superoxide, hydroxyl, and 2,2-diphenylpicrylhydrazyl (DPPH) radicals of EAPS reached 52.39 ± 1.61%, 51.29 ± 1.71% and 89.47 ± 2.89%, which were 44.40%, 49.14% and 33.04% higher than those of APS, respectively (Fig. 3A–C). Furthermore, the higher reducing power could indicate the stronger antioxidant activities. Figure 3D showed that the reducing power of EAPS reached 0.578 ± 0.021, which was 165.14% higher than that of APS (0.218 ± 0.019) at the concentration of 1000 mg/L.

### The serum biochemical assays

**Figure 4:**
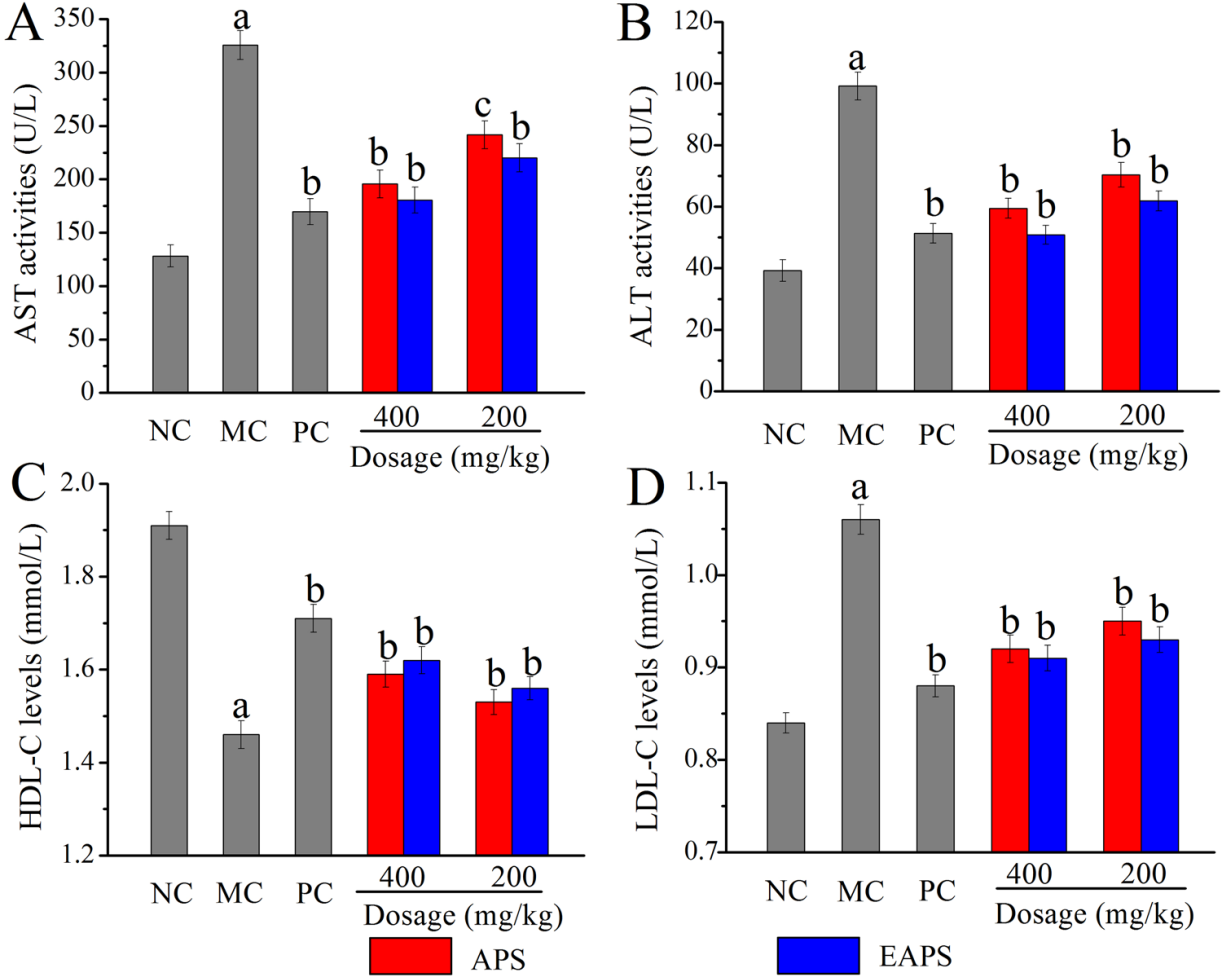
Effects of APS and EAPS on (**A**) AST activities, (**B**) ALT activities, (**C**) HDL-C levels and (**D**) LDL-C levels in serum. The values were expressed as the Mean ± S.D. of five mice per group, (a) *p* < 0.01 compared with NC group, (**b**) *p* < 0.01 compared with MC group, (c) *p* < 0.05 compared with MC group.

Currently, the serum aspartate aminotransferase (AST) activities, alamine aminotransferase (ALT) activities, high-density lipoprotein cholesterol (HDL-C) levels and low density lipoprotein cholesterol (LDL-C) levels could be indirectly reflect the early liver injuries and dyslipidemia. Obviously, the dramatical elevations in activities of AST and ALT were observed by the acute alcohol-injection when compared with that in the alcohol model control (MC) group (*p* < 0.01), indicating that characteristic liver damages had been occurred (Fig. 4A,B). Fortunately, both APS and EAPS had potential effects on decreasing the AST and ALT activities (*p* < 0.05 and *p* < 0.01), especially by the treatment with EAPS at the dose of 400 mg/kg, the AST and ALT activities were reduced to 180.43 ± 12.18 and 50.83 ± 3.09 U/L.

Additionally, significant decrease in HDL-C and remarkable increase in LDL-C levels could be observed in ALD mice when compared with that in the normal control (NC) group, indicating that serious dyslipidemia had been occurred after the alcohol injection (Fig. 4C,D). When treated with EAPS at the dose of 400 mg/kg, the HDL-C levels reached 1.62 ± 0.029 mmol/L, which was higher than that in APS-treated mice (1.59 ± 0.028 mmol/L), while the LDL-C reached 0.91 ± 0.014 mmol/L, which was lower than that in APS-treated mice, demonstrating that both APS and EAPS had effects on improving the lipid metabolism, and the EAPS showed superior effect than APS.

### Effects of APS and EAPS on antioxidant status *in vivo*

**Figure 5:**
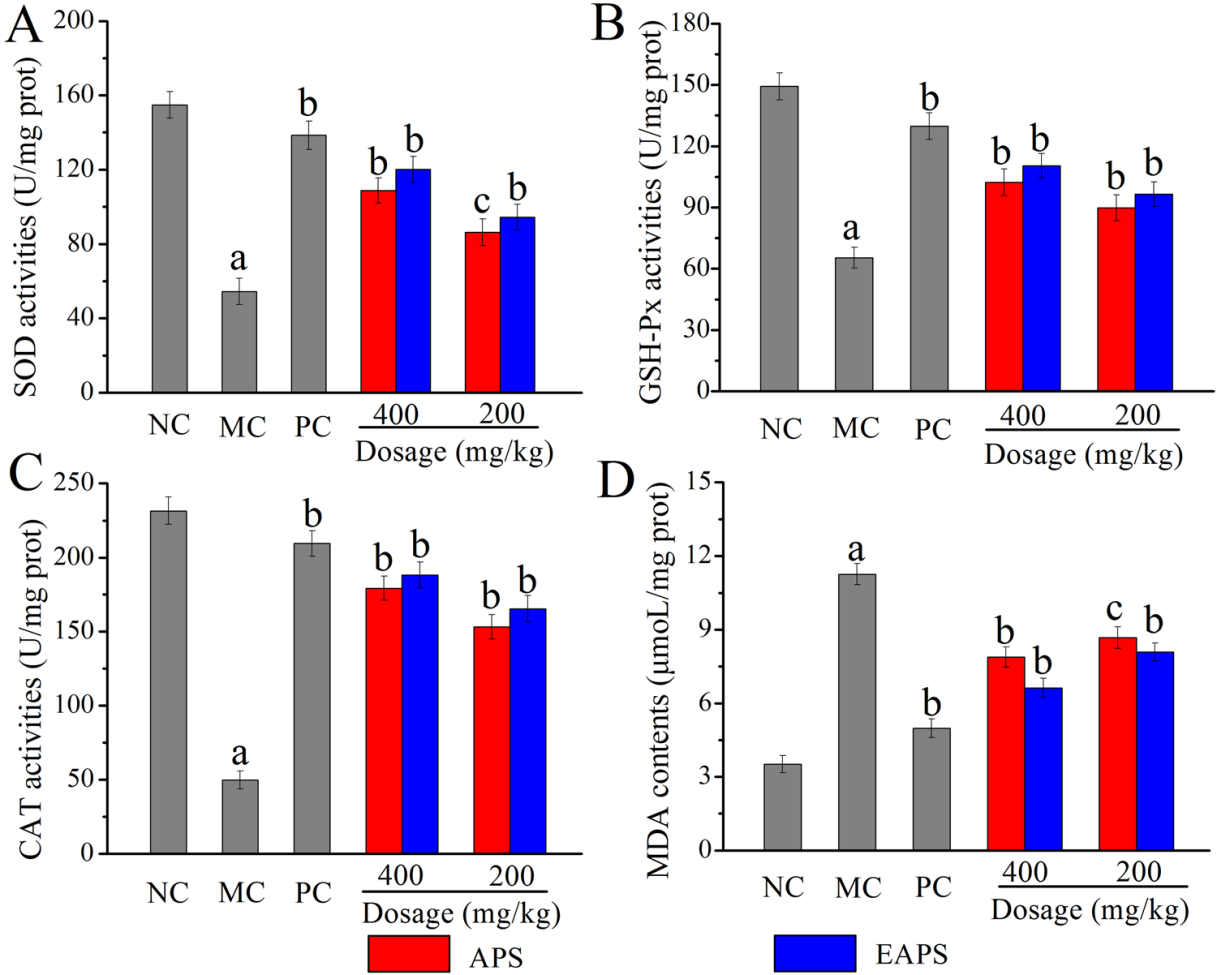
The effects of APS and EAPS on *in vivo* antioxidant status. (**A**) SOD activities, (**B**) GSH-Px activities, (**C**) CAT activities and (**D**) MDA contents. The values were expressed as the Mean ± S.D. of five mice per group, (a) *p* < 0.01 compared with NC group, (b) *p* < 0.01 compared with MC group, (c) *p* < 0.05 compared with MC group.

Enzyme activities of superoxide dismutase (SOD), GSH peroxide (GSH-Px) and catalase (CAT), as well as lipid peroxidation of malondialdehyde (MDA) were applied in evaluating the antioxidant activities *in vivo* (Fig. 5). Obviously, significant decreases in SOD (*p* < 0.01), GSH-Px (*p* < 0.01) and CAT (*p* < 0.01) activities, and significant increases in MDA contents (*p* < 0.01) were observed in ALD mice (MC groups) when compared with that in the NC group, indicating that serious oxidative stress could be induced by acute alcohol injection. Interestingly, the administration of polysaccharides in enhancing the activities of SOD, GSH-Px and CAT, and decreasing the MDA contents could be observed, demonstrating that both APS and EAPS had potential effects on remitting the alcohol-induced oxidative stress. Besides, bifendate-administration (150 mg/kg) could also effectively prevent the decline in hepatic activities of SOD, GSH-Px and CAT, as well as inhibit the rise in MDA contents of bifendate-positive control (PC) group.

### Effects of APS and EAPS on hepatic parameters

**Figure 6:**
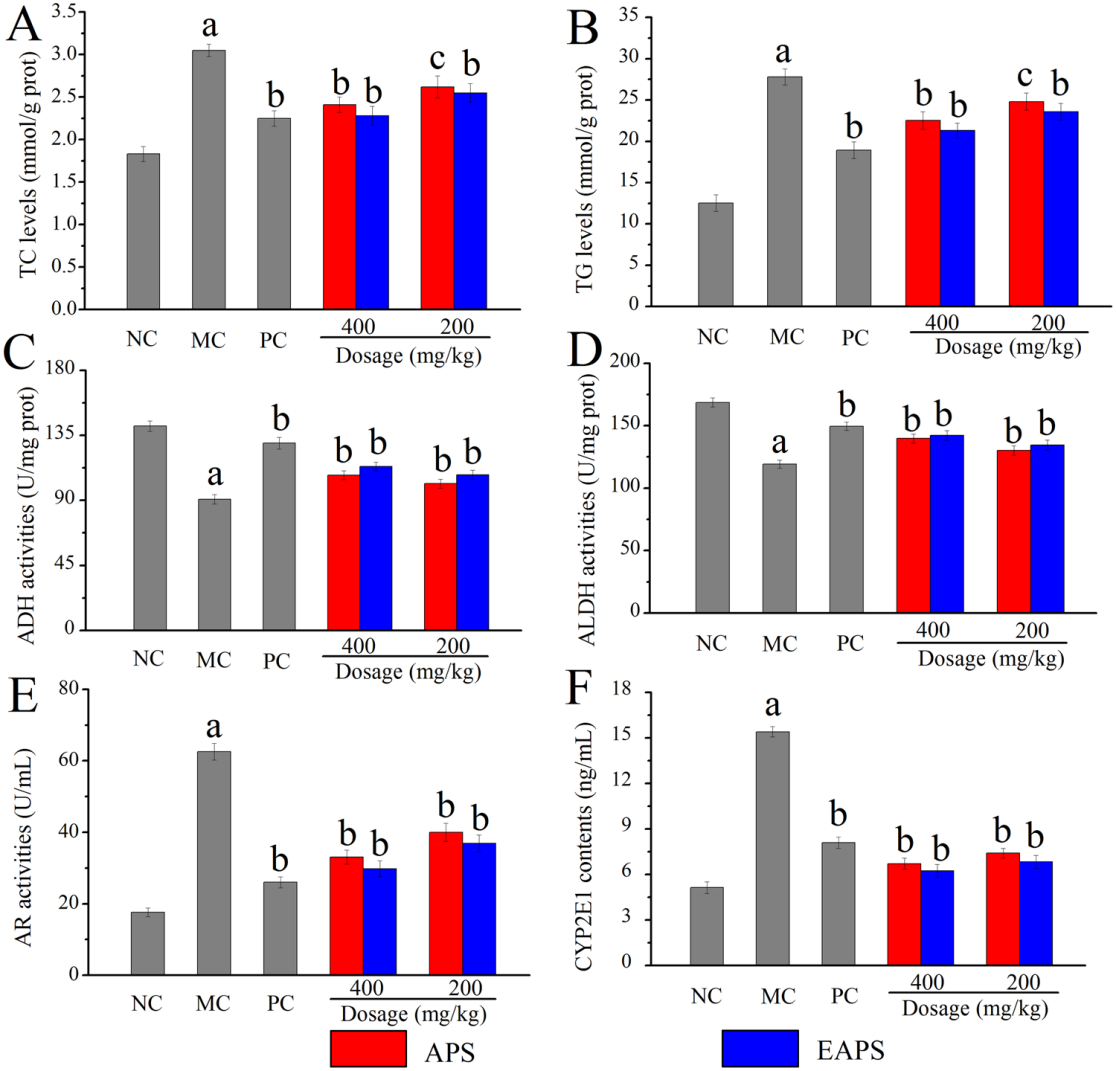
Effects of APS and EAPS on hepatic parameters of (**A**) TC levels, (**B**) TG levels, (**C**) ADH activities, (**D**) ALDH activities, (**E**) AR activities and (**F**) CYP2E1 contents. The values were expressed as the Mean ± S.D. of five mice per group. (a) *p* < 0.01 compared with NC group, (b) *p* < 0.01 compared with MC group, (**c**) *p* < 0.05 compared with MC group.

As shown in Fig. 6A,B, the hepatic levels of total cholesterol TC and triacylglycerols (TG) in ALD mice (MC group, 3.05 ± 0.072 and 27.81 ± 0.98 mmol/g prot) were obviously higher than that in the NC group (1.83 ± 0.089 and 12.53 ± 1.02 mmol/g prot). After the administration of APS or EAPS at different dosages, the hepatic TC and TG levels were significantly reduced (*p* < 0.05 and *p* < 0.01). Especially by the treatment with EAPS at the dosage of 400 mg/kg, the levels of TC and TG were markedly declined to 2.28 ± 0.11 and 21.32 ± 0.89 mmol/g prot, respectively. Meanwhile, the PC group also manifested significantly declined in hepatic TC and TG compared with the MC group (*p* < 0.01).

Additionally, the alcohol dehydrogenase (ADH), aldehyde dehydrogenase (ALDH) and CYP2E1 activities in liver were commonly used in determining the effects of polysaccharides on the alcohol metabolism pathways in reducing liver damages. As illustrated in Fig. 6C,D, the ADH and ALDH activities in alcohol-induced ALD mice (MC group, 90.48 ± 3.24 and 119.27 ± 3.21 U/mg prot) were significantly (*p* < 0.01) lower than that in the NC group (141.32 ± 3.67 and 168.75 ± 3.54 U/mg prot). It is worth noting that ADH and ALDH activities reached 113.28 ± 2.98 and 142.23 ± 3.94 U/mg prot by the treatment with EAPS at a dose of 400 mg/kg, which were markedly (*p* < 0.05) higher than that in the MC group (90.48 ± 3.24 and 119.27 ± 3.21 U/mg prot). As for the activities of aldose reductase (AR) depicted in Fig. 6E, was notably increased (*p* < 0.01) in MC group (62.54 ± 2.36 U/mL), while the AR activities in liver was remarkably decreased (*p* < 0.01) compared with NC group (17.62 ± 1.22 U/mL). After administration of bifendate and two kinds of polysaccharides, the AR activities in dose groups of mice inhibited significantly (*p* < 0.01), indicating that polysaccharides extracted from the *A. cornea* var. *Li*. had potential effects on inhibiting AR activities. Furthermore, as shown in Fig. 6F, the acute alcohol injection could induce the enhancement of hepatic CYP2E1 contents when compared with that in the NC group (*p* < 0.01). Conversely, both APS and EAPS had effects on decreasing the CYP2E1 contents.

### Effect of APS and EAPS on iNOS and COX-2 protein expression

**Figure 7:**
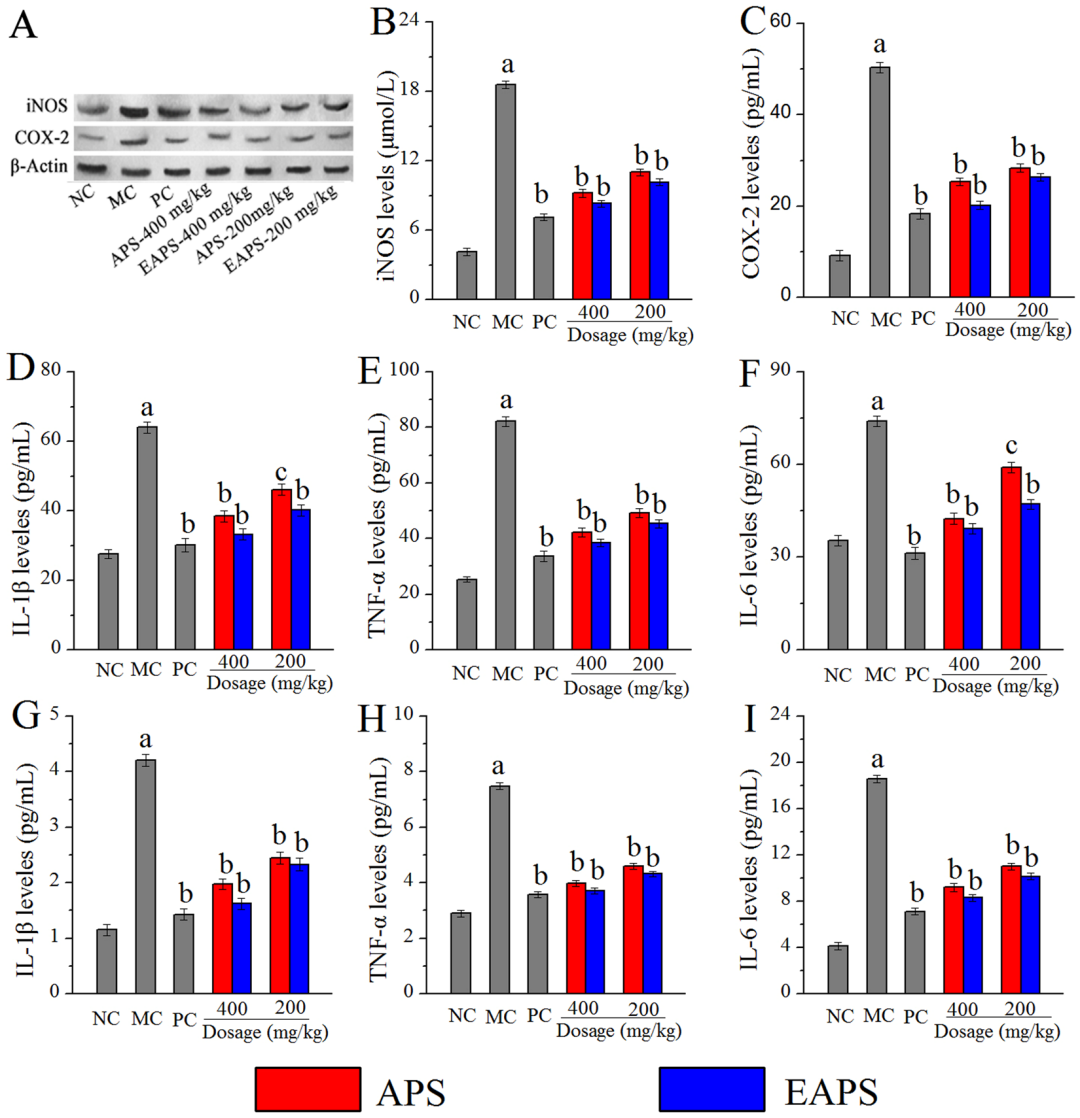
Effects of APS and EAPS on the (**A**) iNOS and COX-2 protein expression, hepatic levels of (**B**) iNOS, (**C**) COX-2, (**D**) IL-1β, (**E**) TNF-α and (**F**) IL-6, and plasma levels of (**G**) IL-1β, (H) TNF-α and (I) IL-6. The values were expressed as the Mean ± S.D. of five mice per group. (a) *p* < 0.01 compared with NC group, (b) *p* < 0.01 compared with MC group, (c) *p* <0.05 compared with MC group.

As shown in Fig. 7A, the protein expression of iNOS and COX-2 were evaluated by western blotting. The alcohol could induce the enhancement of protein expression. Interestingly, the upregulation could be effectively attenuated by oral administration of APS or EAPS, and the dosage of 400 mg/kg showed superior effects. The results demonstrated that the polysaccharides may be favorable towards treating alcohol-induced inflammatory liver damage (The data were in Supplementary File 1).

### Effect of APS and EAPS on inflammatory response

To investigate the effects of ethanol on the inflammatory response, the expression of iNOS, COX-2, IL-1β, TNF-α and IL-6 in liver, and IL-1β, TNF-α and IL-6 in plasma were determined (Fig. 7). The results showed that administration of alcohol promoted (*p* < 0.01) the levels of these inflammatory mediators in mice hepatic homogenate and plasma when compared with that in the NC group. The expression of iNOS, COX-2, IL-1β, TNF-α and IL-6 in liver could be decreased by the treatment of APS or EAPS (*p* < 0.01 or *p* < 0.05) as compared to that in the MC group (Fig. 7B–F). Similarly, APS or EAPS pre-intake had effects on lowering the alcohol-induced increasing of the IL-1β, TNF-α and IL-6 levels in plasma when compared with that in the MC group (Fig. 7G–I). Taken together, these results strongly supported the capability of APS and EAPS on protections against alcohol-induced liver injury by inhibiting pro-inflammatory mediators.

### Histopathological observations

**Figure 8:**
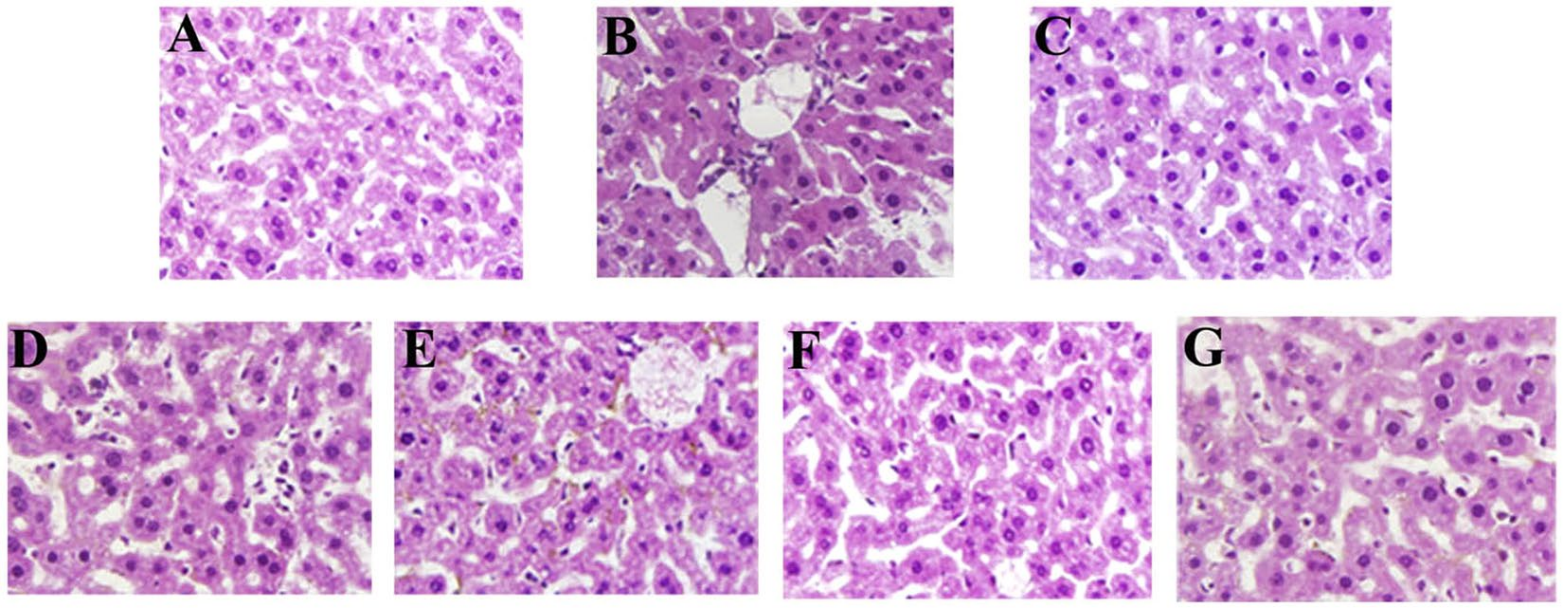
Effects of APS and EAPS on hepatic cells in liver tissue of alcohol-intoxicated mice (magnification 400×). (**A**) NC group, (**B**) MC group, (**C**) PC group, as well as (**D** and **E**) APS and (**F** and **G**) EAPS at 400 and 200 mg/kg, respectively.

The histologic examinations of the liver were presented in Fig. 8. Compared with the regular and orderly architectures of mice in the NC group (Fig. 8A), the alcohol-induced ALD mice showed severe damages including massive fatty changes, incomplete morphology, necrosis, and differed size, *etc*. As shown in Fig. 8D–G, the liver sections of the mice treated with APS or EAPS (400 and 200 mg/kg) showed more or less normal cellular architecture with mild steatosis and necrosis compared to that in the MC group, indicating that both APS and EAPS had potential effects on protection liver tissue against the acute alcohol toxicity. Besides, the bifendate treatment markedly attenuated the ethanol-induced liver damages (Fig. 8C).

## Discussion

Compared with the conventional extraction techniques of maceration, mechanical heat reflux, rabbling, acidic hydrolysis and ultrasound assistance with the defective properties of longer extraction time, high extraction temperature, expensive equipment and environmental pollution, the enzyme assisted extraction offers many advantages such as high extraction yield, simplified manipulation, lower investment costs and less energy requirements^21^. Hence, the enzymatic extraction seems to be an effective and sensible method for extracting polysaccharides^22^. Furthermore, the mice models by acute alcohol administration have experimentally used on investigating the potential hepatoprotective effects of natural substance against the ALD^23^. Besides, previous studies have indicated that the liver damages can be relieved by reducing ROS production^24^. Therefore, the present work was designed to demonstrate the potential antioxidation and hepatoprotection of polysaccharides from *A. cornea* var. *Li*.

ROS, one kind of prooxidants including DPPH, hydroxyl, hydrogen peroxide and superoxide radicals *etc*., is frequently generated spontaneously during the biological metabolism^25^, and palys vital roles in exaggerating the liver diseases^24^. Briefly, the superoxide anions are highly toxic and have been proved to be associated with many diseases^26^. Furthermore, the superoxide anions were commonly considered as precursors of other free radicals, such as the hydroxyl radicals^27^. Therefore, it is essential to remove superoxide anions radicals for the protection of life systems^28^. Meanwhile, the hydroxyl radicals are the most powerful radicals involved in a variety of ROS and dangerous to human health by destroying macromolecules of carbohydrates, lipids, nucleic acids and amino acids^29^. The compounds with good hydroxyl radical scavenging activities show important effects against the oxidative stress^30^. As for DPPH radicals, which are recognized as compounds derived from lipid free radicals inducing oxidative damages in cells, have been widely used to evaluate the antioxidation of various natural products^31^. In addition, the reducing power, reflecting the electronic donation abilities, is often considered as the vital antioxidant ability indicator of biological compounds^32^. Previous research has shown that the AR, a key enzyme in the polyol pathway, which is a monomeric oxidoreductase that catalyses the nicotinamide adenine dinucleotide phosphate (NADPH)-dependent reduction of a wide variety of carbonyl compounds, can be up-regulated in patients with alcoholic liver diseases. However, a dramatic reduction of NADPH resulted in changes in the cellular redox potentials, and further exacerbating intracellular oxidative stress^33^. In addition to oxidative stress, inflammatory injury is also a major property of ALD. It has been reported that the inflammation is a physiological phenomenon on responding the injury, stress and infection, and the inflammatory mediators of iNOS, COX-2, IL-1β, TNF-α and IL-6 can be increased when ALD occurred clinically^34,35^. And studies have demonstrated that alcohol evoked the release of inflammatory cytokines such as IL-1β, TNF-α and IL-6 in the liver, which intensified hepatic inflammation and cell apoptosis. In addition, clinical studies have shown that the plasma concentrations of IL-1β, TNF-α and IL-6 are increased in ALD patients, and the high levels can enhance the severity and mortality^36,37^. On the other side, both iNOS and COX-2 are responsible for the production of large amounts of pro-inflammatory mediators. Thus, inhibition on iNOS and COX-2 levels showed potential effects on improving ALD status^38^. Our present study found that both APS and EAPS had capacities in decreasing the inflammatory levels, providing references on potential development of polysaccharides for anti-inflammation. Furthermore, many evidences have shown that the antioxidant properties of the polysaccharides were mainly connected with the monosaccharide compositions^39^. The present results demonstrated that both APS and EAPS have potential scavenging abilities on these free radicals, indicating that the abundant xylose and glucose probably had potential effects in possessing the antioxidant activities.

Various hepatic transaminases (AST and ALT) could be leaked out of the cytochylema into blood when serious liver damages are occurred, leading the dramatic increases in the serum^40^. Thus, the serum AST and ALT activities are clinically considered as biochemical markers on monitoring the liver damages^41^. The current work showed that both APS and EAPS alleviate the ethanol-induced hepatocellular injuries. As for indicators on monitoring the lipid metabolism, the excess LDL-C in the blood can be easily oxidized, producing foam cells and blood/hepatic patches, while the HDL-C can eliminate surrounding tissue free cholesterol the liver, discharging from the body by bile acids and excretion, hence, elevated LDL-C levels and reduced HDL-C levels are also major factors in aggravating the liver diseases^42^. Therefore, it is essential to improve the lipid metabolism in the prevention and treatment of ALD.

It has been reported that the ALD is associated with the lipid peroxidation, but the exact mechanisms are still unclear^43^. In present work, in order to evaluate the antioxidant activities and hepatoprotective effects of APS and EAPS against ALD, the main antioxidant enzymes and inhibition on lipid peroxidation were measured in liver homogenates, which are considered to be the main defense systems against the oxidative stress^44^. Briefly, the SOD can eliminate superoxide radicals by transforming them in to H_2_O_2_, which can be subsequently decomposed into H_2_O and O_2_ by GSH-Px and CAT, thereby protecting biological membrane against oxidative damage^45^. Moreover, the lipid peroxidation is currently reflected the oxidative stress, and the MDA contents are usually applied in evaluating the antioxidant effects^46^. In present study, both APS and EAPS showed excellent protections against the alcohol-induced ALD contributing to antioxidation activities by preventing the decreases in SOD, GSH-Px and CAT activities, as well as the increases in MDA contents. The present findings were also confirmed by other reports^47^. Additionally, the excessive hepatic TC and TG levels can elevate the endogenous lipid levels, accelerating the progress of ALD^48^. Several pivotal enzymes including ADH, ALDH and CYP2E1 are used for alcohol metabolism. During the metabolism in liver, the ethanol is mainly catalyzed to acetaldehyde by ADH, subsequently oxidized to acetic acid by ALDH. Besides, the micro-alcohol oxidation system dominated by CYP2E1 also plays an important role in ethanol metabolism. It has reported that the CYP2E1 contents could be increased after alcohol management, and during the CYP2E1 metabolism of alcohol, overproduction of ROS could be produced^49^. The present results demonstrated that the management of the polysaccharides could increase the activities of ADH and ALDH, and inhibit the contents of CYP2E1, indicating that both APS and EAPS had potential effect on accelerating the alcohol metabolism, promoting the liver functions.

## Materials and Methods

### Materials and chemicals

The fruiting-bodies of *A. cornea* var. *Li*. were provided by Beijing Academy of Agriculture and Forestry Sciences (Beijing, China). Standard monosaccharide sugars were provided by Merck Company (Darmstadt, Germany). The kits used in the experiments were purchased from Nanjing Jiancheng Bioengineering Institute (Nanjing, China) and Jiangsu Meibiao Biological Technology Company Limited (Jiangsu, China). Anti-iNOS, anti-COX-2 and anti β-actin monoclonal antibody were purchased from Sigma Chemical Company (St Louis, MO, USA). All other chemicals used in the experiment were purchased from local chemical suppliers.

### Preparation of APS and EAPS

The fruiting-bodies were naturally dried and pulverized into powder using a disintegrator (Shanghai, China). The APS and EAPS were separately obtained referencing to the method of Zhao *et al*. and Li *et al*.^50,51^ with slight modifications. The APS and EAPS were extracted with distilled water (1:20, w/v, 90 °C, 5 h) and snailase solutions (0.2%, 1:20, w/v, 37 °C, 4 h), respectively. The supernatants were obtained by centrifugation (3000 rpm, 15 min), and then precipitated with three-fold volumes of ethanol (95%, v/v) overnight (4 °C). After centrifugation at 10000 rpm for 10 min, the precipitates were collected, deproteinized by Sevag^52^, dialyzed against distilled water and lyophilized by vacuum freeze drying to obtain APS or EAPS.

### Monosaccharide compositions analysis

The monosaccharide compositions analysis was processed based on previous method^21^. Monosaccharide components were identified by comparison with standard monosaccharide sugars of rhamnose, fucose, ribose, arabinose, xylose, mannose, galactose and glucose. The molar ratio was carried out by the area normalization method according to the chromatogram.

### Fourier-transform infrared (FT-IR) spectroscopy analysis

The FT-IR spectrum of sample was recorded with an infrared spectrometer (Nicolet 6700, Thermo Fisher Scientific, USA). The samples were grinded with potassium bromide (KBr) and pressed into pellet for spectrometric measurement in the wavenumber range of 4000–500 cm^−1^.

### ^1^H and ^13^C nuclear magnetic resonance spectroscopy (NMR) analysis

The sample was dissolved in deuterated water (D_2_O). The ^1^H and ^13^C NMR spectra were recorded at 25 °C on a Bruker AV-300 spectrometer operating with 300 MHz.

### *In vitro* antioxidant activities

The scavenging abilities on superoxide radicals were processed by the method of Zhang *et al*.^53^ with minor modification. Reactions were carried out in a mixture containing polysaccharides sample (1 mL), Tris/HCl buffer (2 mL, 50 mmol/L, pH 8.2) and pyrogallic acid (0.1 mL, 60 mmol/L) under the conditions of 25 °C for 20 min. Subsequently, the reaction was terminated by adding 50 μL ascorbic acid (5%, w/v). The absorbance was measured at 325 nm, and the scavenging superoxide radicals abilities were calculated by the following formula:

1 $${\rm{Scavenging}}\,{\rm{abilities}}\,( \% )=({{\rm{A}}}_{0}\,-\,{{\rm{A}}}_{1}){/{\rm{A}}}_{0}\times 100$$

where A_0_ was the absorbance of the blank, and A_1_ was the absorbance of the polysaccharide samples.

The scavenging abilities on hydroxyl radicals were measured using the method of Smirnoff *et al*.^54^ with some modifications. Briefly, the reaction mixture contained ferrous sulfate (1 mL, 9 mmol/L), salicylic acid ethanolic solution (1 mL, 9 mmol/L), sample solutions (1 mL, 100–1000 mg/L) and hydrogen peroxide (1 mL, 0.03%, v/v), using the distilled water as blank. The mixture was incubated at 37 °C for 30 min. Finally, the absorbance was measured at 510 nm. The scavenging hydroxyl radicals abilities were calculated as follows:

2 $${\rm{Scavenging}}\,{\rm{abilities}}\,( \% )=({{\rm{A}}}_{0}\,-\,{{\rm{A}}}_{1}){/{\rm{A}}}_{0}\times 100$$

where A_0_ was the absorbance of the blank, and A_1_ was the absorbance of the sample solution.

The scavenging effects on DPPH radicals were measured according to the method of Liu *et al*.^55^ with minor modification. The 2 mL anhydrous ethanol (appending emulsifier) containing DPPH solutions (0.2 mmol/L) was mixed with 2 mL polysaccharide samples (100–1000 mg/L). After shaken vigorously in the dark (25 °C, 30 min), the absorbance was measured at 517 nm using anhydrous ethanol as a blank. The scavenging abilities on DPPH radicals were calculated by the following formula:

3 $${\rm{Scavenging}}\,{\rm{abilities}}\,( \% )=({{\rm{A}}}_{0}\,-\,{{\rm{A}}}_{1}){/{\rm{A}}}_{0}\times 100$$

where A_0_ was the absorbance of the blank, and A_1_ was the absorbance of the tested samples.

The reducing power was assayed according to the reported method^56^ with a slight modification. The reaction mixtures, containing 1 mL polysaccharide samples (100–1000 mg/L), 2.5 mL phosphate buffer (pH 6.6, 0.2 mol/L) and 1.0 mL potassium ferricyanide (1%, w/v), were incubated at 50 °C for 20 min. Subsequently, 1.5 mL trichloroacetic acid solution (10%, w/v) was added. After centrifugation at 1200 rpm for 10 min, 2.5 mL supernatant was mixed with 2.5 mL deionized water and 2.5 mL ferric chloride (0.1%). The absorbance was determined at 700 nm to obtain the reducing power values directly.

### Animal experiment

The male Kunming strain mice (20 ± 2 g, 6–8 weeks old) were purchased from Taibang Biological Company (Taian, China). The mice were maintained under the following standard environmental conditions of temperature of 22 ± 1 °C, humidity of 60 to 65%, and lights on 12 h every day, during which time the mice were free access to sterile food and water. The experiments were conducted in accordance with procedures approved by the Institutional Animal Care and Use Committee of Shandong Agricultural University, and under the Animals (Scientific Procedures) Act 1986 (amended 2013).

After a 4-day acclimatization, all mice were randomly placed into seven groups (five mice in each group) including one NC group, one MC group, PC group (150 mg/kg), two high dose groups and two low dose groups. The mice in dose groups were treated with APS or EAPS at the dose of 400 and 200 mg/kg per day, using bifendate (150 mg/kg) as PC group, as well as physiological saline solution in NC and MC groups as controls, respectively. The whole gavage procedure was lasted for two weeks. On the 15th day, all mice except that in the NC group were given 2 mL of 50% alcohol solutions for establishing the ALD models^57^.

Blood samples were collected in orbital venous plexus and centrifuged (4000 rpm, 10 min) to obtain serum and plasma. The liver was quickly removed, weighed and homogenized instantly in phosphate buffer (0.2 mol/L, pH 7.4, 0 °C) with a proportion of 1:9 (w/v). The homogenate was centrifuged (5000 rpm) at 4 °C for 20 min and the supernatants were stored at 0 °C for further biochemical analysis.

The serum AST activities, ALT activities, HDL-C levels and LDL-C levels were measured by using automatic biochemical analyzer (ACE, USA).

The activities of SOD, GSH-Px, CAT, ADH, ALDH and AR and contents of MDA, as well as the hepatic levels of TC, TG and CYP2E1 were analyzed by means of the diagnostic kits according to the instructions. In addition, the hepatic levels of IL-1β, TNF-α, IL-6, iNOS, COX-2 and AR, as well as the plasma levels of IL-1β, TNF-α and IL-6 were detected by enzyme-linked immunosorbent assay (ELISA) using murine kits according to the instructions.

The liver iNOS and COX-2 protein expressions in each group were determined by using a western blot method^58^. Briefly, samples were applied to SDS-polyacrylamide gel electrophoresis, and transferred to PVDF membrane. After blocking with nonfat milk (5%), the membranes were incubated with mouse anti-iNOS, anti-COX-2 and anti β-actin at 4 °C overnight and then with secondary antibodies for 1 h. Target proteins were visualized with a chemiluminescence kit and analyzed by using an image analyzer (ATTO, Tokyo, Japan).

The formalin-fixed liver tissue was managed by graded alcohols, embedded in paraffin, and then cut into 5 μm thick sections, staining with Haematoxylin and Eosin (HE) dye. Each section was observed by light microscopy (×400 magnifications).

### Statistical analysis

All the experimental data were expressed as the Mean ± Standard Deviations (S.D.). The statistical significance of the differences among groups was analyzed by ANOVA in the SPSS 16.0 statistical package from International Business Machines Corporation (USA). The differences were considered as statistically significant when *p* < 0.05.

## Conclusion

The present work showed that both APS and EAPS by *A. cornea* var. *Li*. exhibited potential effects against the alcohol-induced ALD possibly by enhancing the scavenging abilities on radicals, elevating the antioxidant activities, reducing the lipid peroxidation, inhibiting the expression of inflammatory mediators and promoting the liver functions, demonstrating that the *A. cornea* var. *Li*. could be used explored as natural resources in applying potentially agents for the prevention and alleviation of alcohol-related liver diseases. Study on further structure analysis and structure-function relationships of APS or EAPS is now in progressing.

## Electronic supplementary material

Supplementary information 1
