# Supplementary material for: Hepatoprotective effects of Auricularia cornea var. Li. polysaccharides against the alcoholic liver diseases through different metabolic pathways
Source: Sci Rep. 2018 May 15;8:7574. doi: 10.1038/s41598-018-25830-w (PMC5953970; doi:10.1038/s41598-018-25830-w)

Hepatoprotective effects of *Auricularia cornea* var. *Li.* polysaccharides against the alcoholic liver diseases through different metabolic pathways

*Xiuxiu Wang [1, 2], Yufei Lan [3], Yongfa Zhu [2], Shangshang Li [2], Min Liu [2], Xinling Song [2], Huajie Zhao [2], Weiru Liu [4], Jianjun Zhang [2], Shouxian Wang [1, *], Le Jia[2, *]*

[1] *Institute of Plant and Environment Protection, Beijing Academy of Agriculture and Forestry Sciences, Beijing Engineering Research Center for Edible Mushroom, Key Laboratory of Urban Agriculture (North), Ministry of Agriculture, Beijing, PR China.*

[2] *College of Life Science, Shandong Agricultural University, Taian, 271018, PR China*

[3] *Taian Academy of Agricultural Sciences, Taian, 271000, PR China*

[4] *The Second High School of Taian, Taian, 271018, PR China*

Supplementary information 1

The animal experiment were performed followed our previous methods and assayed the expression of iNOS and COX-2 in liver by western blot. The western blot result was consistent with the ELISA result that has been used in the experiment

Figure S1

The mice in dose groups were treated with the indicated doses of APS and EAPS, using bifendate (150 mg/kg) as PC group, as well as physiological saline solution in NC and MC groups as controls, respectively. (A) Effects of APS and EAPS on the iNOS and COX-2 protein expression in liver, (B) and (C) hepatic levels of iNOS and COX-2. The values were expressed as the Mean ± S.D. of five mice per group. (a) $p < 0.01$ compared with NC group, (b) $p < 0.01$ compared with MC group, (c) $p < 0.05$ compared with MC group.

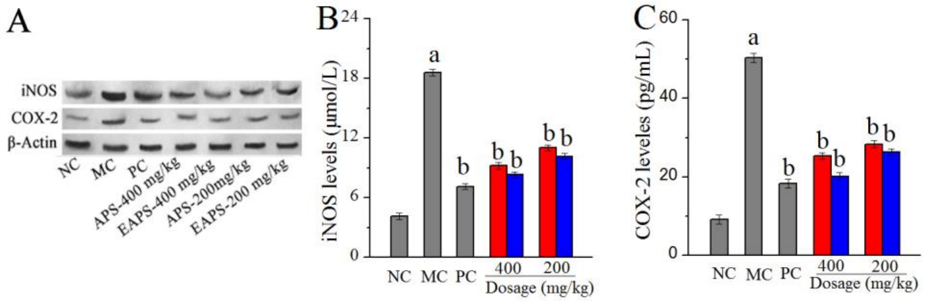

Supplementary information total gel

Figure S2

The iNOS blot cropped from above gel, the COX-2 blot cropped from below gel and the β-Actin used as control.

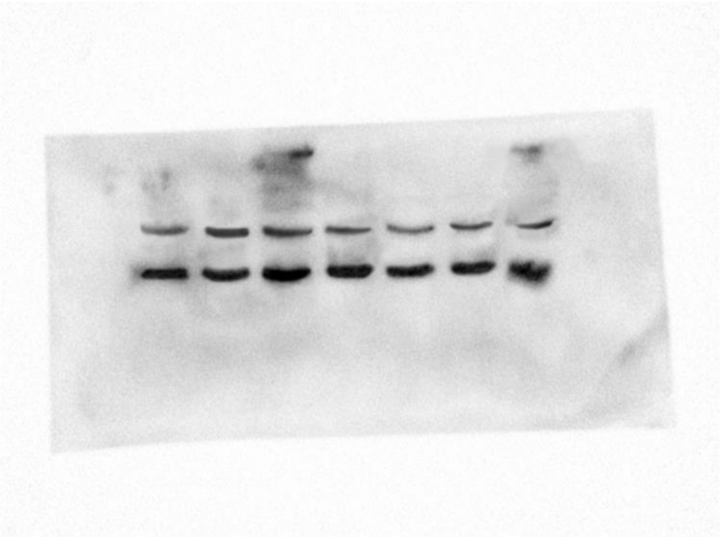

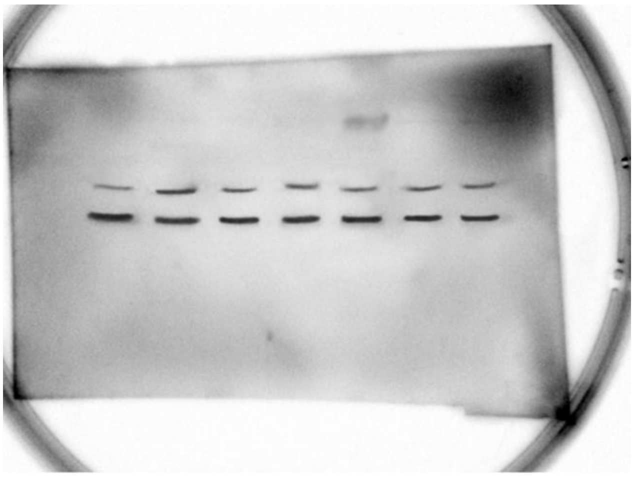

Supplement: Supplementary file 1 — Supplementary information 1 [file 41598_2018_25830_MOESM1_ESM.pdf]
